# The Use of Ovarian Cancer Cells from Patients Undergoing Surgery to Generate Primary Cultures Capable of Undergoing Functional Analysis

**DOI:** 10.1371/journal.pone.0090604

**Published:** 2014-03-06

**Authors:** Rachel L. O′Donnell, Aiste McCormick, Asima Mukhopadhyay, Laura C. Woodhouse, Madeleine Moat, Anna Grundy, Michelle Dixon, Angelika Kaufman, San Soohoo, Ahmed Elattar, Nicola J. Curtin, Richard J. Edmondson

**Affiliations:** 1 Northern Gynaecological Oncology Centre, Queen Elizabeth Hospital, Gateshead, United Kingdom; 2 Northern Institute for Cancer Research, Newcastle University, Newcastle upon Tyne, United Kingdom; 3 Faculty Institute for Cancer Sciences, University of Manchester, Manchester, United Kingdom; H. Lee Moffitt Cancer Center & Research Institute, United States of America

## Abstract

The use of cell lines or animal models has significant disadvantages when dealing with a set of heterogeneous diseases such as epithelial ovarian cancer. This has clinical relevance in that biomarkers developed using cell line or animal models are often not transferable to the clinical setting. In this study, we describe the development of a robust protocol for developing primary cultures of ovarian cancer which will overcome some of these difficulties. Women undergoing surgery for ovarian cancer were recruited and samples of ascites and solid tumour deposits were used to develop primary cultures. Cells were characterised using a panel of immunofluorescent antibodies prior to use in a variety of assays including functional assessment of DNA repair pathways. During the four year study period, viable cultures, confirmed to be epithelial in origin were generated from 156 of 172 (91%) cases recruited. Characterisation was carried out using a panel of antibodies including pancytokeratin, CA125, EpCAM, MOC-31, D2-40 and vimentin. Senescence occurred between the 2^nd^ and 8^th^ passages in all cultures except one in which spontaneous immortalization occurred. Cells could be successfully cultured even after a period of storage at 4°C and cultured cells were capable of being used for a variety of applications including functional assays. Upon functional assessment there was minimal intra-tumour heterogeneity. It is therefore possible to derive viable ovarian cancer cell cultures in the majority of patients undergoing surgery. Cells cultured directly from patient cancers provide an accurate and highly diverse model.

## Introduction

Ovarian cancer is the leading cause of gynaecological cancer mortality worldwide [1] and despite much research into the treatment of ovarian cancer the overall mortality has changed little over the past 20 years with a 5-year overall survival of 30–39% [2]. It has long been recognised by clinicians that ovarian cancer is a set of heterogeneous diseases but despite this ovarian carcinoma continues to be treated clinically as a single disease using a combination of debulking surgery and platinum-based chemotherapy. The observed variation in the clinical behaviour of ovarian cancer alongside the growing data reporting molecular heterogeneity suggests that a heterogeneous model for the study of ovarian cancer is long overdue. The emerging concept of personalised medicine based upon biomarkers of response to novel treatments targeting specific defects in tumour DNA repair is only possible if biomarkers can be tested using a realistic model.

Established cell lines provide an invaluable tool for studying biological functions at the molecular and cellular level. Existing human ovarian cancer cell lines possess the advantage of high proliferative capacity, clonogenecity and extended life span in culture. However, most have acquired significant genetic alterations from their cells of origin, including deletion of important regulatory cell cycle genes supporting immortality. Additionally, there is evidence to suggest that many cell lines contain significant misidentification, duplication, and loss of integrity [3].

Primary cells isolated from patients are often considerably different from established cell lines of similar origin. The ability to culture and characterise freshly isolated OSE (ovarian surface epithelium) and EOC (epithelial ovarian cancer) cells from patients provides an important experimental system that has the potential to resemble the patient situation more accurately [4], [5].

There are two sources of clinical material which have been used to generate primary cultures in ovarian cancer: ascitic fluid and solid tumour tissue. Gene expression studies have indicated different biological profiles in the cancer cells derived from these two sources from the same patient in terms of metastasis, invasion and angiogenesis [6]. Ascitic fluid has several advantages over solid tumour tissue in generating primary cultures. Ascitic fluid is relatively easy to obtain and culturing the suspended cells is technically straight forward. Ascitic cultures have been shown to generate a homogeneous epithelial cell rich population compared to those obtained from solid tissues. Significant proportions of patients with ovarian cancer present at an advanced stage and have large volumes of ascitic fluid which can be obtained during surgery or paracentesis. However, as the majority of patients with large volume ascites have tumours of a high grade serous histological subtype, only sampling ascites will underrepresent the other histological subtypes. Culture of solid tumour, particularly in the absence of ascites is therefore also required to capture a representative group of samples.

Primary cell culture from either source could provide a resource for testing the molecular profile and performing functional studies of individual cancers. In recent years the association between tumour molecular heterogeneity, survival and/or response to treatment has been acknowledged [7] and has fuelled the search for biomarkers to predict response to novel therapies targeting DNA repair pathways deregulated in ovarian cancer.

Several methods for the culture of primary ovarian cancer cells isolated from ascites have been described [8]. These methods however require complex multi-step procedures. Dunfield *et al* and Shepherd *et al* describe a more simple and reliable culture method involving mixing ascites directly with medium which results in epithelial cell culture [4], [9]. This technique has been adapted by our group for use in research into the functional status of DNA repair mechanisms and here we report our experience of this technique.

## Methods

### Ethics statement

The study was approved by local ethics committee (UK IRAS North West ethics committee - 12/NW/0202) and all patients gave written informed consent.

### Reagents

Rucaparib was a gift from Clovis (USA) and is a potent inhibitor of PARP-1 and −2 proteins (with an inhibition constant of <5 nM). All other chemicals and tissue culture reagents were from Sigma-aldrich (Sigma-aldrich, UK), unless otherwise stated.

### Cell Culture

#### Sample collection

Ascites and solid tissue was collected from consented patients undergoing surgery for ovarian cancer at the Queen Elizabeth Hospital, Gateshead, UK. Clinical details were recorded and specimens registered and handled in accordance with the Human Tissue Act. Samples were assigned a PCO (Primary Culture Ovary) reference number to retain anonymity.

#### Sample transport and preparation

Ascites was aspirated directly from the patient into a sterile suction bottle. Solid tumour was placed into a sterile universal containing culture medium (RPMI 1640 medium supplemented with 20% FCS, 20 mM L-glutamine and 1% penicillin and streptomycin) pre-warmed to 37°C. Samples were transported from the hospital to the lab immediately in compliance with UK Category B regulations UN3373.

#### Primary Culture from ascites

Cell culture was performed using an aseptic technique in a containment level II laminar flow microbiological safety cabinet. 20 ml of ascites was added to 20 ml of warmed culture medium (RPMI with 20% FCS, as above) in T75 flasks (Corning, USA) and incubated at 37°C, 5% CO_2_, 95% humidified air. The medium was aspirated and 13 ml of warmed fresh medium was replaced on day 3 to 5. The medium was replaced every 4 to 5 days until the cells approached confluence. Cells were passaged, frozen and thawed as previously described [10]. Cell cultures were quarantined in a primary incubator to await formal pathological examination results and to ensure that infected samples were not introduced into general culture.

#### Primary culture from solid tumour

Once in the laboratory, the solid tumour was dissected into ∼3 mm^3^ pieces using a sterile scalpel and transferred to T25 flasks containing sufficient collagenase/dispase (Roche, UK) solution (1 mg/1 ml in full medium) to fully immerse the sample. The cells were incubated for 2 hours at 37°C on an orbital shaker (IKA-Vibrax-VKR) at 2×G. The cell suspension was transferred to a universal container, centrifuged at 400×G for 5 minutes, PBS washed, re-suspended in full medium and placed in a T25 flask for 30 minutes to allow fibroblast seeding. The epithelial cell suspension was transferred to a T25 flask for on-going cell culture.

### Culture optimisation

#### Direct culturing onto coverslips

Time from collection to functional assessment can be up to 14 days. To reduce the length of culture and handling prior to use, media and ascitic fluid (1∶1 v∶v) was added directly onto sterile cover slips for immediate analysis.

#### Cytospinning

Ascitic fluid was cytospun directly onto microscope slides after optimisation of centrifugation speed, sample volume and enrichment of ascitic fluid using the addition of red cell lysis buffer. Following cytospining, slides were air dried, fixed with 100% methanol and stored at −20°C for later characterisation.

### Characterisation

Ovarian cancer is a set of heterogeneous diseases. Additionally, ascitic fluid contains a variety of cell types and a single marker is therefore insufficent to reliably differentiate epithelial ovarian cancer cells from other cell types. A characterisation panel consisting of cell culture morphology, immunofluorescent staining of fixed cells, as well as standard pathological and immunohistochemistry examination was combined to ensure accurate epithelial characterisation of every culture.

#### Morphology

Morphological features were studied under an Olympus CK40 inverted microscope at 20× magnification. Images were captured using VisiCam software (VWR, USA).

#### Immunofluorescence

Standard techniques for immunofluorescence were used to stain for pancytokeratin, epithelial cell adhesion molecule (EpCAM), cancer antigen 125 (CA125), epithelial related antigen (MOC-31), D2-40 and vimentin, Table 1. None of the positive markers are uniformly expressed in all EOC cells but interpreted in combination enable the selection of appropriate cultures.

**Table 1:** Antibodies used for cell characterisation.

Marker	Description	Concentration	Company
Pancytokeritin	PCO samples were classified to be epithelial in origin if more than 95% of cells stained with mouse monoclonal anti-pancytokeratin FITC–conjugated antibody	1∶100	Upstate Millipore Corp., USA
EpCAM	Epithelial origin was confirmed with positive staining for mouse monoclonal anti-CD326 Alexafluor 488–conjugated antibody	1∶100	Biolegend, USA
(CA125	CA125 is expressed in 80% of epithelial ovarian cancers [22]. Expression was assessed using mouse monoclonal anti-CA125 antibody and Alexafluor 546 goat anti-mouse secondary antibody.	1∶100 (Primary)	Abcam, USA
MOC-31	MOC-31 is present on most normal and malignant epithelia [23] enabling discrimination from mesothelial derived tumours. Expression was assessed using mouse monoclonal anti-MOC-31 antibody and Alexaflour 596 goat anti-mouse secondary antibody.	1∶100 (Primary)	Dako, Germany
D2-40	Anti-D2-40 identifies an O-linked sialoglycoprotein present on germ cell tumors but not epithelial cells [24]. Expression was assessed using mouse monoclonal anti-D2-40 antibody and goat anti-mouse Alexafluor 596 secondary antibody	1∶100 (Primary))	Dako, Germany
Vimentin	Marker commonly used to detect epithelial-mesenchyman transition (EMT). Expression was assessed using rabbit monoclonal anti-vimentin antibody, clone EPR3776 and Alexafluor 488 goat anti-rabbit secondary antibody	1∶100 (Primary)	Abcam, USA

### Formal histopathology

Formal pathological and cytological examination of ascitic and solid tumour specimens from patients donating PCO samples was performed and used to further characterise the cultures.

When all characterisations were in keeping with epithelial ovarian origin, the samples were then used in subsequent experiments. Where results were inconsistent with epithelial origin, cultures were discarded.

### Imagestream^X^ characterisation

#### Established PCO cultures and fresh ascites

PCO cells, cultured using the methods above, were trypsinised, fixed with 0.4% paraformaldehyde for 20 minutes at 4°C, and permeabilised with BD Phosflow Perm/wash I (BD Biosciences, USA; 1∶10 v∶v distilled water).

For fresh ascites, ten ml of ascitic fluid was filtered to exclude large debris (180 µm pore nylon filter, Millipore, UK). As ascitic fluid is frequently contaminated with blood, the addition of a fixative containing red cell lysis buffer (1∶5 v∶v BD Phosflow Lyse/Fix red cell lysis buffer, BD Biosciences, USA) enabled depletion of red blood cells. Cells were permeabilised with BD Phosflow Perm/wash I and the sample further enriched by depletion of white blood cells using EasySep Human CD45 Depletion Kit (STEMCELL technologies, France), as per manufacturer's instructions.

All samples were then incubated with immunoflourescent–labelled antibodies for 12 hours at 4°C including pancytokeratin, EpCAM, CA125, DRAQ5 nuclear stain and common leucicytoe antigen (CD45) to identify white blood cells. Samples were processed using Imagestream^X^ (Amnis, USA) with IDEAS software utilised to quantify the proportion of cells expressing the three epithelial markers, as well as providing an assessment of co-expression.

### Growth and Cytotoxicity assays

#### SRB

A routine sulforhodamine B (SRB) assay was used to assess cytotoxicity and cell growth as previously described [11]. Briefly, cells were seeded at a concentration of 1000 cells/well and after adherence, treated with various concentrations of rucaparib for 10 days before fixation, staining and spectrophotometer assessment.

#### Clonogenic Assays

Clonogenic assays are considered gold standard for the assessment of cytotoxicity. 50,000 cells/well were seeded in a 6 well plate for 24 hours. Cells were then incubated for 24 hours with various concentrations of the cytotoxic agent before reseeding at concentrations of 2,500, 5,000 and 10,000 cells for each drug treatment. Cells were incubated at 37°C for 14 days. The medium was aspirated, plates were washed in PBS and then fixed using the Carnoy's fixative (acetic acid: methanol 1∶3 v/v) followed by staining with 1% crystal violet.

#### Agar clonogenics

50,000 cells were seeded into wells containing media and treated as above for 24 hours in agarose media. Cells were then trysinised, washed and re-seeded in semi-solid agarose (Promega, UK) media, at a variety of concentrations, and incubated for 14 days. Colonies were stained using 3-(4,5-Dimethylthiazol-2-yl)-2,5-diphenyltetrazolium bromide (MTT).

### Cell transfection

Luciferase expressing plasmid pGL2 (Promega, USA) was transfected using two methods. Firstly, the manufacturer's protocol (Invitrogen, USA) for transfecting cells with lipofectamine TM LTX with Plus reagent was used to transfect PCO cultures with 1–6 µg of DNA per well. Secondly, 250 µl of PCO cell suspension containing 1×10^6^ cells/ml was mixed with 1–5 µg of pGL2 plasmid in 4 mm electroporation cuvettes (Eurogentec, Belgium). Electroporation was carried out using an EPI-2500 electroporator at 100–500 volts. The transfectants from both methods were harvested 48 hours after transfection and assayed for luciferase activity.

Furthermore PCO cultures were transfected using MISSION™ shRNA lentiviral transduction particles (Sigma-aldrich, USA), as per manufacturer's protocol.

### Homologous recombination assay

Cells were seeded onto glass cover slips and treated with 2Gy ionising radiation and rucaparib at 10 µM concentration for 24 hours to induce double strand breaks (DSB). All experiments were performed alongside untreated controls with equivalent 0.1% DMSO. Cells were then fixed and rehydrated prior to staining with 1∶100 mouse monoclonal anti-γH2AX (Upstate, Millipore Corp., USA) and 1∶100 goat polyclonal anti-Rad51 (Calbiochem, EMD Biosciences, Inc., USA) antibodies with appropriate secondary fluorochrome conjugated antibodies, as previously described [5].

Image J counting software [12], [13] was used to count γH2AX and Rad51 nucleic foci. Cells were classed as homologous recombination (HR) competent if there was more than a 2 fold increase in Rad51 foci after DNA damage, confirmed by a 2 fold increase in γH2AX.

## Results

### Generation of primary cultures from ascites

**Figure 1:**
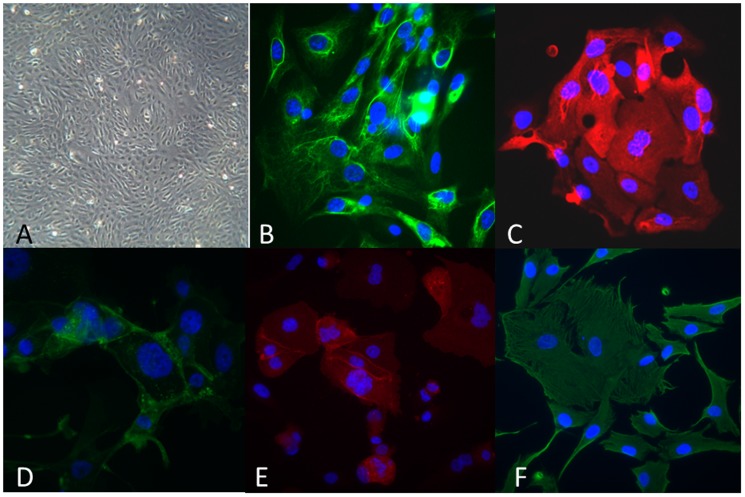
Phenotypic appearances of primary cultures. A: Brightfield demonstrating cobblestone monolayer; immunoflourescent images with antibodies targeted against: B: FITC-anti-pancytokeratin; C: Alexafluor 596 anti-CA125; D: Alexflour 488 anti-EpCAM; E: Alexafluor 596 anti-MOC 31; F: Alexaflour 596 anti-Vimentin

Ascites was collected from 172 ovarian cancer patients undergoing primary (67%) or delayed primary surgery (following three to four cycles of platinum-based neo-adjuvant chemotherapy; 33%) between 2008 and 2013. Viable cultures were generated in 166/172 (97%) cases. Erythrocytes and cellular debris from ascites did not adhere to the culture flask and were removed following media change. In general the appearance of each culture was that of a cobblestone monolayer pattern, Figure 1 (A), as described by Dunfield *et al* [4]. Ascitic fluid is composed of multiple cellular components and in order to confirm exclusive growth of cancer cells characterisation of cultured cells is required. Immunoflourescent characterisation of the cultures was carried out using the panel of antibodies to detetct expression of pancytokeratin CA125, EpCAM, MOC-31, D2-40 and Vimentin, Figure 1 (B-F). 10/166 (6%) were rejected as they failed to demonstrate greater than 95% cytokeratin positivity. The overall success rate therefore for creating ascitic epithelial primary cultures from patients undergoing surgery was 156/172 (91%). Routine histopathological examination of FFPE tissue from each patient was carried out, Table 2. The most predominant histological subtype was high grade serous cancer.

**Table 2:** Patient demographics by histological subtype.

Histological subtype	n =	Median age at diagnosis (years)	Median CA125 (U/ml)	FIGO Stage (%)				Remaining disease >1 cm following surgery (%)
				1	2	3	4	
High grade serous	83	67	1439	1	1	79	19	18
Clear cell	4	67	187	0	0	67	33	0
Endometrioid	7	64	896	29	0	57	14	14
Carcinosarcoma	5	65	618	0	0	60	40	0
Mucinous	3	58	539	100	0	0	0	0
Low grade serous	5	75	1171	0	0	100	0	20
Dysgerminoma	1	26	90	100	0	0	0	0
Borderline tumour	1	66	180	100	0	0	0	0
Other	14	59	190	0	33	50	17	14

**Figure 2:**
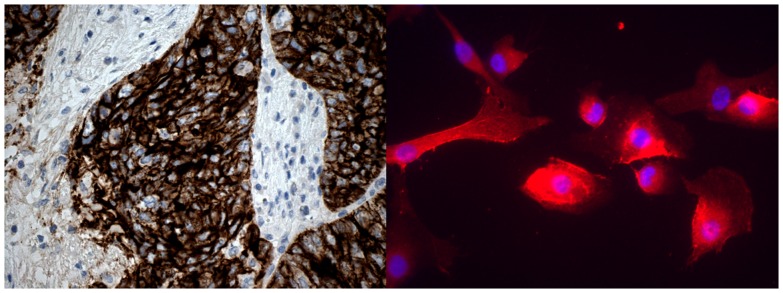
Antigen expression in primary cultures. PCO 158 A: Immunohistochemical detection of CA125 in FFPE tissue; B: Immunofluourescent detection of CA125 with Alexaflour596 from corresponding primary culture

In a subset of samples, immunoflourescent assessment of CA125 expression in the PCO sample was compared with the CA125 expression on immunohistochemical analysis of FFPE tissue from the same patient, Figure 2. Correlation of results from the two assessments was seen in 8/11 (72%) cases. In the remaining three cases, two showed IHC expression of CA125 only, whilst one showed IF expression only.

Cellular morphology was studied over time and it was seen that the majority of cultures were of cobblestone morphology but late passage cells (typically following passage 4/5) developed a more mesenchymal phenotype, becoming elongated and exhibiting a markedly reduced growth rate. Senescence occurred between the 2nd and 8th passages, most commonly between 4th and 5^th^, thus rendering further characterisation at late passage impossible. Cultures were considered unsuccessful when no growth was seen after 28 days. One culture spontaneously immortalized and has been grown to more than 20 passages. It is not clear why culture from ascites is unsuccessful in a proportion of cases, why senescence occurs at variable passages or why only one culture has immortalised. It is likely however that this is a consequence of a lack of essential factors required for growth which are provided *in vivo* by the complex interactions within the tumour microenvironment and which are lacking in the artificial culture environment.

### Culture growth

**Figure 3:**
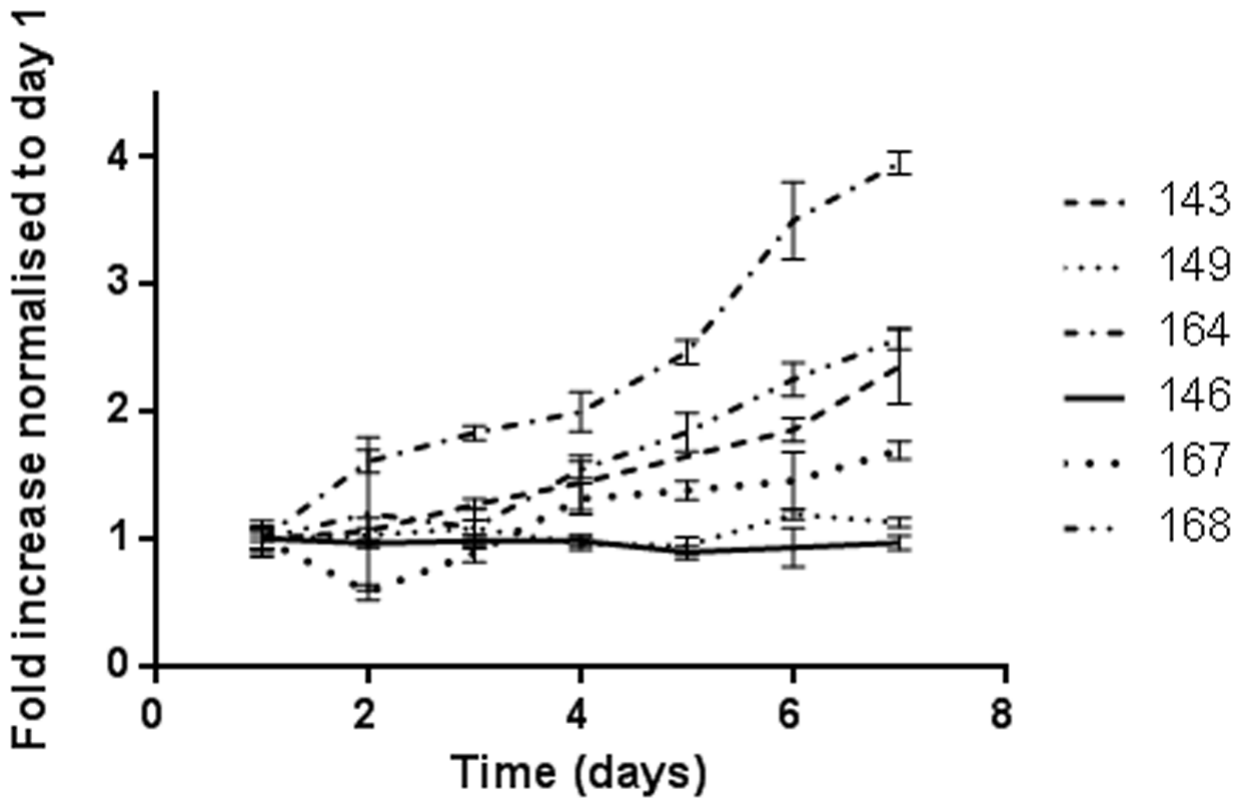
Growth curves for 6 representative PCO cultures (PCO143, 146, 149, 164,167 and 168). The mean and SD of 6 repeats for each time point is plotted, normalised to day 1.

Growth rates of 30 PCO cultures, grown in RPMI media supplemented with 20% FCS, were measured by SRB assays using early passage cells to generate doubling times. Doubling times between PCO cultures were highly variable with a median doubling time of 100 hours (range 79 to 195 hours), Figure 3. However, cells from the same culture tested at different passage showed minimal variability in doubling time (mean difference of 0.9 hours; SEM 4.8). No correlation was demonstrated between growth rate, histological subtype or stage of disease at presentation. The relatively slow growth rate seen may also be a consequence of the articial culturee environment and lack of factors from the tumour micronvironment.

### Primary culture from solid tumour

**Figure 4:**
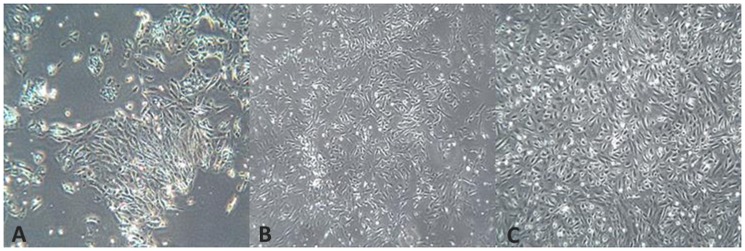
Brightfield images of PCO 163 which was derived from solid tissue explant. A: Passage 0 at 48 hours; B: Passage 0 at 72 hours; C: Passage 1 at 14 days

**Figure 5:**
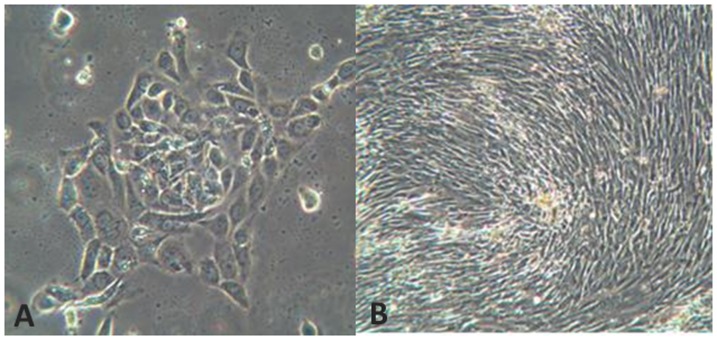
Brightfield images of PCO 162 demonstrating the effects of selective seeding. A: Epithelial cell growth from supernatant removed 30 minutes after initial plating (×20 magnification); B: Cells adhering during the initial 30 minute incubation were almost entirely fibroblastic (×10 magnification).

In parallel with ascites, solid tissue was collected from 11 patients and processed as described. Establishment of cell cultures from tissue explants was achieved in 100% of cases. The explant morphology was lost 72 hours post-seeding as cells acquired a monolayer cobblestone appearance with time and passaging. Cultures showed a similar morphological appearance to those derived from ascitic cell culture, Figure 4. Fibroblast contamination was minimised by plating the cells for 30 minute incubation before removing the epithelial rich supernatant and replating, Figure 5.

### Sample transport

#### Transport optimization

Place of sample collection is frequently distant from laboratory facilities. In order to use this model in collaborative translational work successful establishment of culture following transport needs to be considered. We therefore compared the success of culture characterization and functional assays following various transport methods.

**Group 1 - Fresh ascites (n = 10):** transport to the lab with processing within 6 hours of collection, using the method described above.

**Group 2 - Room temperature samples (n = 7):** ascites was stored at room temperature in a sterile container for 24 hours before mixing 1∶1 with media in a flask as above.

**Group 3 - Refrigerated samples (n = 10):** ascites was stored at 4°C for 24 hours before mixing 1∶1 with medium as above.

**Group 4 - Frozen samples (n = 6):** paired sets of 50 ml aliquots of ascitic fluid were centrifuged at 400G for 5 minutes. The resultant cell pellets were frozen, stored at −80°C and −120°C and thawed at 6 weeks and 6 months.

Successful cultures were attained from all transport groups however in cases with delayed culture it was preferable to maintain cultures at 4°C. There was no difference in morphology of the cultures grown from each group, Table 3.

**Table 3:** Primary culture outcomes following transport.

Sample transport			n =	Culture success	
				Passage 1	Passage 2
Group 1 -Fresh collection			75	70 (90%)	64 (85%)
Group 2 - Room temperature			7	5 (71%)	4 (57%)
Group 3 –4 °C			10	9 (90%)	9 (90%)
Group 4 -Frozen	6 weeks	−80°C	6	6 (100%)	6 (100%)
		−120°C	6	6 (100%)	6 (100%)
	6 months	−80°C	6	0	0
		−120°C	6	5 (83%)	5 (83%)

Ascitic cell pellets (Group 4) stored at −80°C and −120°C were thawed at 6 weeks and 6 months. Cultures were successfully grown from both storage conditions after 6 weeks with no difference observed in morphology, growth rate or functional assessment. However, following 6 months storage, a significant difference in success of subsequent culture from the two conditions was observed. Ascitic pellets stored at −120°C were successfully cultured in 83% cases. No cultures stored at −80°C could be successfully grown.

### Coverslip cultures

The morphological appearance of coverslip cell cultures was akin to parallel cultures processed using the standard method. Immunoflourescent characterisation was consistent across all PCO cases between coverslip and traditional method cultures. Optimal immunoflourescent staining for characterisation was obtained if performed more than 24 hours after seeding.

### Cytospinning

The optimum parameters to balance maximal cell adherence with preservation of nuclear morphology were 200 µl of ascitic fluid (concentration of 10×10^4^ cells per ml) cytospun at 80×G for 5 minutes. Despite steps taken to deplete the ascitic fluid sample of unwanted non-epithelial cells and debris, slides remained heavily contaminated, confirmed by sub-optimal cytokeratin staining and poor CA125 expression. Contamination made assessment of morphology, immunoflourescent characterisation and functional assessment of HR status unsuccessful.

### Imagestream^X^ assessment of PCO cultures

The introduction of Imagestream^X^ technology with its ability to combine flow cytometry with high resolution immunoflourescent microscopy permitted the assessment of expression of a number of immunofluorescent-labelled markers within individual cells simultaneously at high speed and high resolution. Immunoflourescent detection of the three epithelial ovarian markers (pancytokeratin, EpCAM and CA125), assessed using standard fixed immunofluorescence and Imagestream^X^ technology was consistent in 13/15 cases (87%), Table 4. In addition Imagestream^X^ enabled the quantification of percentage expression and assessment of co-expression.

**Table 4:** Comparison between the characterisation results from immunoflourescent microscopy (IF) and Imagestream^X^ (IS^X^).

Sample	EpCAM		CK		CA125	
	IF	IS^x^	IF	IS^x^	IF	IS^x^
**PCO 1**	Negative	Negative	Positive	79% Positive	Variable	11% Positive
**PCO 2**	Negative	Negative	Positive	98% Positive	Variable	4% Positive
**PCO 3**	Positive	Negative	Positive	90% Positive	Positive	28% Positive
**PCO 4**	Negative	Negative	Positive	84% Positive	Variable	13% Positive
**PCO 5**	Variable	10% Positive	Variable	35% Positive	Variable	36% Positive

### Imagestream^X^ assessment of fresh ascites samples

**Figure 6:**
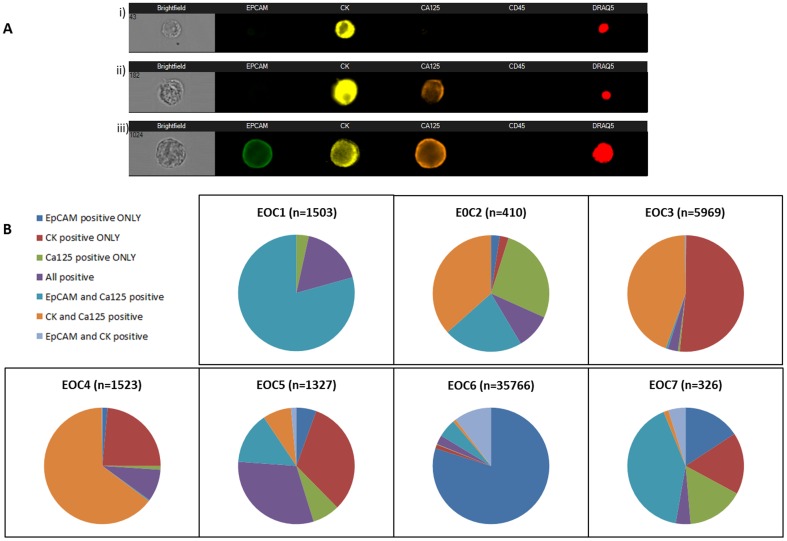
A: ImagestreamX cell plot of representative cells demonstrating the three major cellular sub-populations within ascitic fluid, ; i) CK positive only, ii) CK and CA125 positive and iii) EpCAM, CK and CA125 positive. B: Sub-populations identified in the epithelial cell population in EOC ascitic fluid (n = the total number of epithelial cells identified in 10 ml of ascites).

Ten ml of fresh ascites was collected and analysed from seven ovarian cancer patients using Imagestream^X^. Cells were classified as epithelial if they were nucleated, negative for CD45 and positive for EpCAM, CK or CA125. The epithelial cell population in each ascitic sample could be divided into a number of sub-populations based upon the pattern of antibody labelling, Figure 6a and b, and demonstrated great inter-tumour heterogeneity. The proportion of cells staining positive for EpCAM was highly variable with a median of 52% (0–98%). There was no correlation between the Imagestream^X^ determined expression of epithelial markers and the status as determined by fixed immunofluorescence (ROC curve, not shown, AUC of 0.5). It is possible that some of these subpopulations may represent non-epithelial cells (ie mesothelial cells) but it also possible that they are in fact epithelial cells and undergo phenotypic change as a result of the culture method; or that the culture method positively selects out subpopulations. Cell sorting with subsequent molecular profiling of each of the subpopulations would clarify this and enable further molecular differences in subsets of EOC to be explored.

### Cell transfection

A number of functional assays require transfection of vectors into cells [14], [15]. Despite optimisation, both lipofectamine and electroporation transfection methods failed to yield a high enough transfection efficiency for functional assays.

However, cells continued to grow in puromycin media following transfection using MISSION™ shRNA lentiviral transduction particles suggesting successful transfection. Long term transfection could not be assessed due to the short term life-span of the PCO cultures.

### Functional homologous recombination assays and cytotoxicity

The RAD51 assay for HR DNA repair status was successfully performed in all primary cultures which were grown successfully from all transport groups. HR status for cultures from the same PCO donor using direct plating onto coverslips and ascitic cultures were all consistent. Culturing directly onto cover slips reduced the time taken from collection to determine HR status from approximately 14 to 5 days.

As expected the GI_50_ values following treatment with rucaparib for each PCO culture were variable, however a clear differentiation of sensitive and resistant samples was seen as previously described [5]. In the majority of cases, as expected, PCO samples with defective HR status were sensitive to rucaparib, whilst those with competent HR status were resistant, Table 5.

**Table 5:** HR DNA repair status of PCO culture with corresponding sensitivity to 10 µM Rucaparib.

PCO culture	HR status		Sensitivity to Rucaparib*	
	Group 1 Fresh collection	Group 4 Frozen	Group 1 Fresh collection	Group 4 Frozen
58	+	+	R	R
61	+	+	R	R
63	+	−	R	R
66	+	+	R	R
69	+	+	R	R
87	+	+	R	R
88	+	+	R	R
91	+	+	R	R
60	−	−	S	S
67	−	−	S	S
68	−	−	S	S
75	−	−	S	S
77	−	−	S	S
89	−	−	S	S
90	−	−	S	S

*Cell survival following 10 µM Rucaprib. Resistant (R)  =  more than 70% survival; Sensitive (S)  =  less than 70% survival.

Despite optimisation of several standard clonogenic techniques, colony formation was not seen and therefore clonogenic assays abandoned in PCO cultures. Cell line work has previously demonstrated a linear relationship between results obtained from SRB and clonogenics and therefore SRB was adopted as the standard technique in this study [16].

## Discussion

The ability to generate and utilise primary cultures of ovarian cancer has several advantages over other models including established cell lines and animal models. It is now recognised that many cell lines in long term culture will undergo further genetic aberrations rendering them dissimilar from their tissue of origin. Furthermore even a large panel of cell lines cannot recapitulate the tremendous heterogeneity that is seen in epithelial ovarian cancer. Many animal models are merely xenografts utilising these cell lines and therefore retain the same set of problems. Transgenic mouse models tend to have been generated by integrating a small number of mutations [17] and therefore do not display the massive chromosomal instability which is a recognised hallmark of ovarian cancer [18].

Here we have described our own findings for generating primary cultures of ovarian cancer, using cells derived from both ascites and solid deposits of disease. The ability to culture from both of these materials is important as, dependent upon the clinical setting, only one of these materials may be available. For instance, in relapsed disease it is not unusual to see ascites in the absence of large solid deposits in contrast to the initial presentation setting where only solid disease may be present. We have reported a sequential set of samples to demonstrate the success rate for generating cultures. 156/172 (91%) samples collected resulted in a viable culture of epithelial cells. This figure is sufficiently high to justify the feasible use of these techniques in routine clinical practice if diagnostic tests were developed for use with this material.

Often, and indeed in our own case, there may be significant geographical separation between the operating room and the diagnostic laboratory. The ability to transport samples without loss of integrity is therefore essential and we have described techniques demonstrating that these samples can safely be maintained at either 4°C or −20°C for up to 24 hours before culturing. Furthermore, the ability to store cultures long term in liquid nitrogen allows the possibility of collaboration for research or *post hoc* diagnostic analysis.

Particularly at high stage when tumours are disseminated throughout the abdominal cavity and beyond, many tumours will demonstrate significant intra-tumour heterogeneity [19], [20] although often these changes are more related to local stromal reaction than to the presence or absence of key driver mutations [21]. The ability to culture both ascites and solid tissue derived cells allows investigation of intra-tumour heterogeneity although so far our results suggest that both morphology and outcome of functional assays is similar in the different subcultures taken from the same patient. This suggests that HR function is a global characteristic of the cancer, independent of heterogeneity and therefore may potentially reflect mutations resulting in dysfunctional HR status as an early event that has driven the cancer formation.

One of the strengths of developing models of viable cancer cells is that it allows for the use of functional assays which would not be possible using FFPE tissue or even fresh frozen tissue. This is likely to become increasingly important in the development of biomarkers for treatments which depend upon the dysregulation of a complete pathway as opposed to aberration of a single gene.

In conclusion therefore we have described the techniques for collecting, transporting and culturing cells from patients with ovarian cancer. We believe this to be safe and reliable and in our opinion is a useful way of generating tissue for use both in translational studies and potentially for functional diagnostic testing.

## References

[1] Statistics NOf (2011) Mortality statistics: Deaths registered in 2010 (Series DR) Table 5.2. National Office for Statistics.

[2] Parmar MK, Ledermann JA, Colombo N, du Bois A, Delaloye JF, et al (2003) Paclitaxel plus platinum-based chemotherapy versus conventional platinum-based chemotherapy in women with relapsed ovarian cancer: the ICON4/AGO-OVAR-2.2 trial. Lancet 361: 2099–2106. 12826431 10.1016/s0140-6736(03)13718-x

[3] Korch C, Spillman MA, Jackson TA, Jacobsen BM, Murphy SK, et al (2012) DNA profiling analysis of endometrial and ovarian cell lines reveals misidentification, redundancy and contamination. Gynecol Oncol 127: 241–248. 22710073 10.1016/j.ygyno.2012.06.017 PMC3432677

[4] Dunfield LD, Shepherd TG, Nachtigal MW (2002) Primary culture and mRNA analysis of human ovarian cells. Biol Proced Online 4: 55–61. 12734568 10.1251/bpo34 PMC145557

[5] Mukhopadhyay A, Elattar A, Cerbinskaite A, Wilkinson SJ, Drew Y, et al (2010) Development of a functional assay for homologous recombination status in primary cultures of epithelial ovarian tumor and correlation with sensitivity to poly(ADP-ribose) polymerase inhibitors. Clin Cancer Res 16: 2344–2351. 20371688 10.1158/1078-0432.CCR-09-2758

[6] Le Page C, Ouellet V, Madore J, Ren F, Hudson TJ, et al (2006) Gene expression profiling of primary cultures of ovarian epithelial cells identifies novel molecular classifiers of ovarian cancer. Br J Cancer 94: 436–445. 16421595 10.1038/sj.bjc.6602933 PMC2361148

[7] Hennessy BT, Murph M, Nanjundan M, Carey M, Auersperg N, et al (2008) Ovarian cancer: linking genomics to new target discovery and molecular markers-the way ahead. Adv Exp Med Biol 617: 23–40. 18497028 10.1007/978-0-387-69080-3_3 PMC2844243

[8] Hirte HW, Clark DA, Mazurka J, O′Connell G, Rusthoven J (1992) A rapid and simple method for the purification of tumor cells from ascitic fluid of ovarian carcinoma. Gynecol Oncol 44: 223–226. 1531803 10.1016/0090-8258(92)90046-l

[9] Shepherd TG, Theriault BL, Campbell EJ, Nachtigal MW (2006) Primary culture of ovarian surface epithelial cells and ascites-derived ovarian cancer cells from patients. Nat Protoc 1: 2643–2649. 17406520 10.1038/nprot.2006.328

[10] Lee EC (1991) Cytogenetic analysis of continuous cell lines In The ACT Cytogenetic Laboratory Manual: 107-148.

[11] Vichai V, Kirtikara K (2006) Sulforhodamine B colorimetric assay for cytotoxicity screening. Nat Protoc 1: 1112–1116. 17406391 10.1038/nprot.2006.179

[12] Abramoff M, Magelhaes P, Ram S (2004) Image processing with ImageJ. Biophoton Int 11: 36–42.

[13] Znojek P (2011) PhD Thesis. Newcastle University.

[14] Bau DT, Mau YC, Ding SL, Wu PE, Shen CY (2007) DNA double-strand break repair capacity and risk of breast cancer. Carcinogenesis 28: 1726–1730. 17494053 10.1093/carcin/bgm109

[15] Ohashi A, Zdzienicka MZ, Chen J, Couch FJ (2005) Fanconi anemia complementation group D2 (FANCD2) functions independently of BRCA2- and RAD51-associated homologous recombination in response to DNA damage. J Biol Chem 280: 14877–14883. 15671039 10.1074/jbc.M414669200

[16] Perez RP, Godwin AK, Handel LM, Hamilton TC (1993) A comparison of clonogenic, microtetrazolium and sulforhodamine B assays for determination of cisplatin cytotoxicity in human ovarian carcinoma cell lines. Eur J Cancer 29A: 395–399. 8398340 10.1016/0959-8049(93)90394-u

[17] Fong M, Kakar S (2009) Ovarian cancer mouse models: a summary of current models and their limitations. J Ovarian Res 2. 10.1186/1757-2215-2-12 PMC2762470 19781107

[18] Bowtell DDL (2010) The genesis and evolution of high-grade serous ovarian cancer. Nat Rev Cancer 10: 803–808. 20944665 10.1038/nrc2946

[19] Khalique L, Ayhan A, Weale ME, Jacobs IJ, Ramus SJ, et al (2007) Genetic intra-tumour heterogeneity in epithelial ovarian cancer and its implications for molecular diagnosis of tumours. The Journal of Pathology 211: 286–295. 17154249 10.1002/path.2112

[20] Abelson S, Shamai Y, Berger L, Shouval R, Skorecki K, et al (2012) Intratumoral Heterogeneity in the Self-Renewal and Tumorigenic Differentiation of Ovarian Cancer. STEM CELLS 30: 415–424. 22267284 10.1002/stem.1029

[21] Kobel M, Turbin D, Kalloger S, Gao D, Huntsman D, et al (2011) Biomarker expression in pelvic high-grade serous carcinoma: comparison of ovarian and omental sites. Int J Gyn Path 30: 366–371. 10.1097/PGP.0b013e31820d20ba 21623201

[22] Rosen DG, Wang L, Atkinson JN, Yu Y, Lu KH, et al (2005) Potential markers that complement expression of CA125 in epithelial ovarian cancer. Gynecol Oncol 99: 267–277. 16061277 10.1016/j.ygyno.2005.06.040

[23] Souhami RL, Beverley PC, Bobrow LG, Ledermann JA (1991) Antigens of lung cancer: results of the second international workshop on lung cancer antigens. J Natl Cancer Inst 83: 609–612. 2023279 10.1093/jnci/83.9.609

[24] Marks A, Sutherland DR, Bailey D, Iglesias J, Law J, et al (1999) Characterization and distribution of an oncofetal antigen (M2A antigen) expressed on testicular germ cell tumours. Br J Cancer 80: 569–578. 10408868 10.1038/sj.bjc.6690393 PMC2362349

